# Removal of the Thyroid Gland

**Published:** 1878-01

**Authors:** Julius F. Miner


ART. III.—*Removal of the Thyroid Gland.* By Prof. JULIUS F. MINER, M. D. Reported by CHAS. A. WALL, B. S.

In a previous number of this Journal (Vol. XVI. No. 12), Dr. Miner presented an article upon the *Feasibility of Extirpating the Thyroid Gland in some Cases of Disease, with report of a case* in which he took occasion to say, "that some goitrous tumors may be extirpated with the knife, if other means fail, with *reasonable hope of success.*" It is with great pleasure that we communicate the following case which occurred in the Doctor's practice since the above was written, because it corroborates the views there stated.

Mr. J. B. K., German, a baker by trade, aged 42, called at the office in order to obtain relief from a tumor which troubled him. He had been under treatment for considerable time by some of the best physicians but without relief. Iodine externally with iodide of potassium internally had been used to produce absorption; indeed, every medicine supposed to have beneficial effect upon glandular enlargements had been tried, and as a last measure he was advised to place himself under surgical treatment. Upon examination, the tumor was found to be hard and but slightly movable. It lay upon the trachea and occupied the region of the thyroid body which it was decided to be. It was more developed upon the left than upon the right side, but this was not apparent except upon close and careful examination; and the upper left angle seemed to be more firm than the same portion on the right side. This was found, after removal, to have been caused by a deposit of calcarious material. He gave the following history:

When about nineteen years of age a slight swelling was noticed which gradually enlarged but after a time it diminished in size but never entirely disappeared; it remained stationary but increased in solidity. This did not cause any considerable inconvenience until within about three years when he first noticed difficulty in respiration after exercise or when stooping. This increased, and for over a year past he would waken at night with a feeling of suffocation. It became so distressing that, as he expressed it, "he often wished to die at once and be done with it rather than live to die every night." The dangers of the operation were explained to him and he was advised to wait until he could no longer endure it. Electrolysis was used, not with any hope that it would disperse the tumor, for it was almost impossible to introduce the needles, but for the purpose of gratifying him. He soon became impatient and desired relief saying that he would gladly take all risks, anything being preferable to the suffering he then experienced. Seeing that he would not be put off, his request for surgical aid was granted and Oct. 25th, Dr. J. F. Miner operated for the removal of the tumor with the assistance of Drs. W. W. Miner, O'Brien, J. C. Greene and Brush. A few medical students were also present.

The patient having been brought under the influence of Ether an incision three inches in length was made diagonally across the tumor, which was then siezed with strong tenaculum forceps and raised from its bed. As soon as this was done the patient who before had struggled for breath began to breathe easier. The tumor was now enucleated the vessels being tied as approached, some of them before division. After the first incision the knife was discarded, the dissection of the attachments being accomplished by the handle of a scalpel. There was considerable hemorrhage which was controlled by ligature without the use of the cautery. The arterial supply was from the usual branches but they were only to be known from their situation and not from their size or importance. No artery was found of the size of the superior thyroid, as supplying the gland in health. The firmest attachment was at the base being the entrance of the inferior arteries, which were ligated and divided. The wound was closed with superficial sutures and water dressings applied. He rallied immediately from the operation, had no trouble with it afterwards, and he suffered so little that the necessity of keeping his head still could not be impressed upon him. In due course of time the ligatures came away and the wound healed; in two weeks he was at work feeling better and able to do more than he had for three years. There was an entire absence of any bad symptoms.

The tumor removed consisted of a hard mass having the appearances of cartilage in which were found small calcarious deposits with two or three cysts in the center of the body. It was irregularly quadrilateral in form the corners being rounded; the anterior surface was convex, while the posterior was slightly concave having half an inch to the right of the center a perpendicular groove, half an inch deep and three-quarters of an inch wide, marked transversely by the rings of the trachea. Its weight was ℥vj, ʒiv, gr xv. It was nine (9) inches in circumference, three and three-quarters ($3\frac{3}{4}$) inches in breath, four (4) inches in length, and two and one-half ($2\frac{1}{2}$) inches in thickness. The union of the two lobes could not be made out for the neck had so enlarged as to entirely fill up the interval between them. From its position, size, appearance and the relation of other structures to it, there can be no doubt but that there was in this case an enlargement and solidification of the thyroid gland of slow growth. The pressure upon the trachea was so great that it flattened it and misled one physician who made a laryngiscopical examination and induced him to state that the impediment to respiration was due not to the pressure from the enlarged gland but to a small tumor which was growing upon the interior of the trachea, but that he had no instruments by which it could be removed.

The points of greatest interest are the facility with which the gland was removed, and the comparatively slight hemorrhage which was so easily controlled. This was owing to the greatly lessened vascular supply, due no doubt to the nature of the degeneration; and the case is confirmatory of the statement made in the article previously quoted, "that a diseased gland may in some cases be more safely extirpated than a healthy one."

